# Study of spectral overlap and heterogeneity in agriculture based on soft classification techniques

**DOI:** 10.1016/j.mex.2024.103114

**Published:** 2024-12-17

**Authors:** Shubham Rana, Salvatore Gerbino, Petronia Carillo

**Affiliations:** a Department of Engineering, University of Campania “L. Vanvitelli”, Via Roma 29, Aversa 81031, CE, Italy; b Department of Biological and Pharmaceutical Environmental Sciences and Technologies, University of Campania “L. Vanvitelli”, Via Antonio Vivaldi, 43, Caserta 81100, CE, Italy

**Keywords:** Soft-classification, Fuzzy logic, Digital image processing, Chlorophyll absorption ratio index (CARI), Soil adjusted vegetation index (SAVI), Modified soil adjusted vegetation index (MSAVI), Modified possibilistic C-means (MPCM), Intra-class Heterogeneity mapping in Agriculture

## Abstract

This study explores the application of fuzzy soft classification techniques combined with vegetation indices to address spectral overlap and heterogeneity in agricultural image processing. The methodology focuses on the integration of three key vegetation indices: Soil-Adjusted Vegetation Index (SAVI), Modified Soil-Adjusted Vegetation Index (MSAVI), and Modified Chlorophyll Absorption in Reflectance Index (MCARI), with Modified Possibilistic C-Means (MPCM) clustering. The analysis involves preprocessing the image data, calculating the vegetation indices, and applying the MPCM algorithm to perform soft classification, allowing pixels to belong to multiple classes with varying degrees of membership. A quantitative assessment is conducted to evaluate the accuracy of the classification results. Methodological approach:

• Integrating advanced image processing techniques and vegetative band ratios with the fuzzy classification method MPCM to handle the inherent complexities in agricultural image analysis, such as spectral overlap and mixed boundaries.
• Quantitative assessment of classification accuracy using Fuzzy Error Matrices (FERM).

This approach provides a robust framework for analyzing spectral overlaps among the crops and weeds and improving the accuracy of crop classification, particularly in heterogeneous environments.

## Graphical abstract

**Figure:**
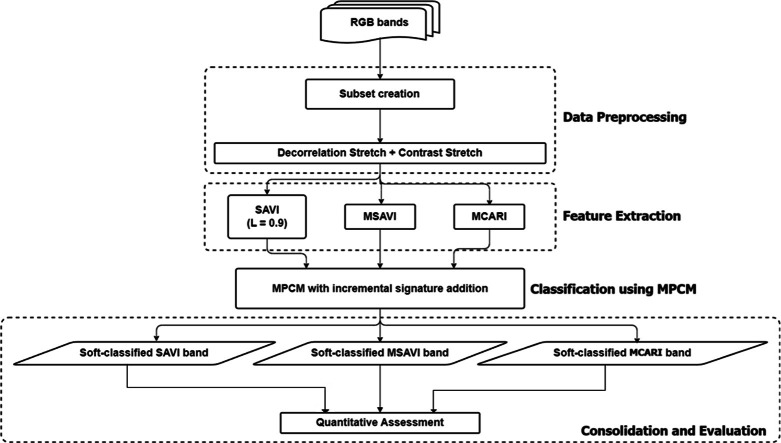

Specifications table

**Table:** 

Subject area:	Engineering
More specific subject area:	Digital Image Processing
Name of your method:	Intra-class Heterogeneity mapping in Agriculture
Name and reference of original method:	Not Applicable
Resource availability:	https://data.mendeley.com/datasets/dcjjcwc5dh/3

## Background

The methodology detailed in this study is driven by the need to address the complexities inherent in agricultural image processing, particularly in heterogeneous environments where spectral overlap and variability are significant challenges. Precision agriculture relies heavily on accurate classification and segmentation of crops, weeds, and soil to inform management and decision-making. However, traditional classification techniques often struggle with the natural variability in spectral signatures, leading to issues like spectral similarity between crops and weeds, which often results in classification inaccuracies [[1], [2], [3], [4]].

One of the major challenges is the presence of mixed pixels, where a single pixel contains information from both crops and weeds, making it difficult for fuzzy-based algorithms to distinguish between them accurately. Additionally, varying environmental conditions—such as differences in lighting, soil backgrounds, and growth stages—further complicate the accuracy of spectral indices and challenge the consistency of fuzzy algorithms across different scenarios [[5], [6], [7], [8]].

The high dimensionality of hyperspectral data, which is often used alongside fuzzy algorithms, increases computational complexity and poses challenges for real-time applications due to the significant computational resources required [9,10]. Selecting the most relevant spectral indices and tuning fuzzy algorithm parameters is a complex task, as different agricultural contexts may yield varying results, necessitating extensive experimentation and calibration [[11], [12], [13]]. Furthermore, integrating spatial and spectral information to enhance classification accuracy presents additional challenges, requiring sophisticated algorithms and precise calibration [14,15].

To overcome these challenges, this study integrates fuzzy soft classification techniques with vegetation indices to handle the nuances of spectral overlap and variability. The combination of Modified Possibilistic C-Means (MPCM) clustering with indices like the Soil-Adjusted Vegetation Index (SAVI), Modified Soil-Adjusted Vegetation Index (MSAVI), and Modified Chlorophyll Absorption Ratio Index (MCARI) offers a more robust approach. These indices were chosen for their ability to account for soil brightness and chlorophyll content, which are crucial in distinguishing between crops and weeds in heterogeneous environments.

The MPCM algorithm's ability to assign varying degrees of membership to pixels allows for a more nuanced classification, addressing the problem of mixed pixels and providing a more accurate representation of the actual conditions in the field. Additionally, the use of soft classification ensures that transitions between different land cover types are more realistically modeled, reducing the likelihood of abrupt class changes that are not reflective of the real-world scenario.

In conclusion, while the combination of vegetation-based spectral indices with fuzzy-based algorithms holds promise for crop and weed classification and segmentation, addressing issues of spectral similarity, environmental variability, occlusion, high dimensionality, optimal index selection, and spatial-spectral integration requires further research. This study's methodology is motivated by the potential to improve accuracy and reliability in these areas, enhancing decision-making processes in precision agriculture [[16], [17], [18], [19]]. The dataset of which this research

## Method details

### Overview

This study employs a comprehensive methodology to handle spectral overlap and heterogeneity in agricultural image processing, aiming to improve the accuracy of crop and weed classification and segmentation in heterogeneous agricultural environments. The approach integrates fuzzy soft classification techniques with vegetation indices, addressing the challenges posed by spectral similarity, environmental variability, and computational complexity. The subsequent operations were performed on a curated dataset which is now manually as well as automatically annotated [20].

#### Image acquisition and preprocessing

**Fig. 1:**
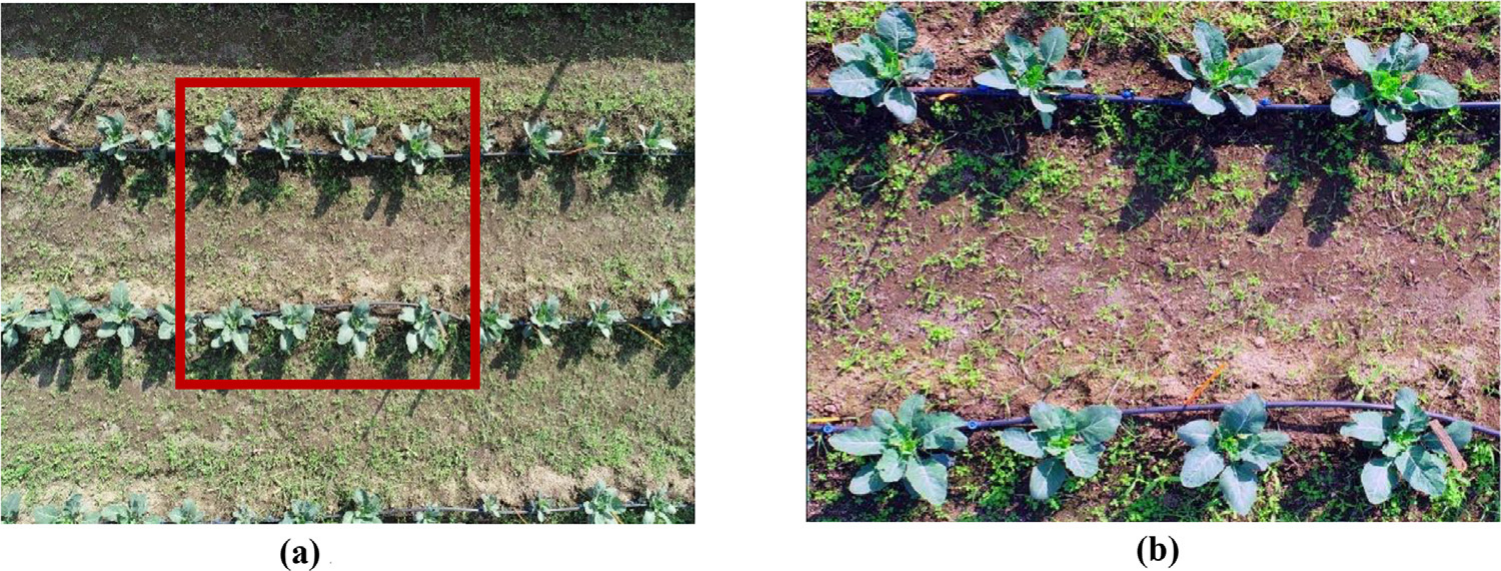
**(a)** Aerial image with subset marked in red rectangle **(b)** Preprocessed subset with application of decorrelation stretch and contrast stretch.

The initial step (Fig. 1) involves acquiring RGB bands from aerial sensing data, which represent the red, green, and blue channels essential for various imaging and aerial sensing-based applications [2]. After acquiring these bands, subsets are created from the aerial images to focus on a specific area of interest within the larger dataset, thereby improving the efficiency and relevance of the analysis [7]. To enhance the visual separability of features, two key preprocessing techniques are applied (Fig. 1(b)):

**Decorrelation Stretch**: It is an image enhancement technique commonly used in remote sensing and digital image processing to improve the visual distinction between features in an image by enhancing colour contrast. This technique is especially useful for images where subtle colour differences make it difficult to distinguish between features, such as satellite or aerial imagery. Often, natural images have correlated colour bands (Red, Green, Blue) due to the inherent properties of the surfaces being imaged and the way light interacts with them. This correlation can obscure subtle differences between features, making it challenging to identify specific areas of interest.

The decorrelation stretch process begins with transforming the original RGB colour bands into an uncorrelated colour space using principal component analysis (PCA) or a similar statistical method. PCA decorrelates the colour channels by aligning the axes with the direction of maximum variance in the data, effectively reducing the correlation between the colour bands. Once transformed, each principal component is independently stretched, expanding the range of values to use the full dynamic range of the display device, typically from 0 to 255 in an 8-bit image. This stretching step significantly increases the contrast of features within each principal component. After this, the image is transformed back to the original RGB colour space. The result is an image with enhanced colour separation, making previously subtle features more visible and easier to interpret [[2], [21]].

**Contrast Stretch**: It is also known as contrast enhancement and is a fundamental image processing technique used to improve the visibility of features in an image by expanding the range of intensity values. This technique adjusts the contrast of an image by stretching the histogram of pixel intensity values to occupy a wider range of the available intensity scale, typically from the minimum to the maximum possible values (e.g., 0 to 255 in an 8-bit image). The goal of contrast stretching is to enhance image features that are not easily distinguishable due to low contrast, making them more prominent and easier to analyse.

The contrast stretch process begins by identifying the minimum and maximum intensity values in the image. These values define the range over which the image's pixel intensities are currently distributed. The next step involves linearly scaling these values so that the minimum intensity is mapped to 0 (the darkest possible value), and the maximum intensity is mapped to 255 (the brightest possible value). All other pixel values are adjusted proportionally within this new range. This technique adjusts the image contrast, increasing the visibility of features by expanding the dynamic range of pixel values. This enhancement is critical for improving boundary clarity, ensuring that the classification process accurately identifies different regions within the image [4]. Mathematically, the contrast stretch can be represented as (Eq. 1):

(1) $$I^{\prime }=\;\frac{\left(I-I_{min}\right)}{\left(I_{max}-I_{min}\right)}\;\times \left(I_{new\_max}-I_{new\_min}\right)\;+\;I_{new\_min}$$

Where:

• **I** is the original pixel intensity
• **I'** is the new pixel intensity after stretching
• **I_{min}** and **I_{max}** are the minimum and maximum pixel intensities in the original image
• **I_{new_min}** and **I_{new_max}** are the desired minimum and maximum intensities in the output image.

#### Vegetation Indices Calculation

The next step involves calculating three vegetation indices from the pre-processed images:

**Soil-Adjusted Vegetation Index (SAVI)**: The Soil-Adjusted Vegetation Index (SAVI) is a modified form of the widely used Normalized Difference Vegetation Index (NDVI), designed to minimize the influence of soil brightness, which can significantly affect vegetation indices, especially in areas with sparse vegetation. The SAVI was introduced to address this issue by incorporating a soil brightness correction factor (L) into the traditional NDVI formula, allowing for more accurate vegetation assessments in environments where the soil surface is exposed.The SAVI formula (Eq. 2) is expressed as follows [22]:

(2) $$SAVI=\;\frac{\left(NIR-Red\right)\left(1+L\right)}{\left(NIR+Red+L\right)}$$

A common value for L is 0.5, but it can be adjusted based on the amount of vegetation cover. SAVI is particularly useful in semi-arid and arid regions where the soil background significantly affects vegetation indices [23].

**Modified Soil-Adjusted Vegetation Index (MSAVI)**: The Modified Soil-Adjusted Vegetation Index (MSAVI) is an enhancement of the Soil-Adjusted Vegetation Index (SAVI), specifically designed to further reduce the influence of soil background on vegetation indices, particularly in areas with low vegetation cover [23]. While SAVI introduced a correction factor (L) to adjust for soil brightness in the calculation of vegetation indices, MSAVI refines this approach by making the correction factor dynamic, allowing it to adapt based on the amount of vegetation present in the pixel. The MSAVI is calculated using the following formula (Eq. 3):

(3) $$MSAVI=2NIR+\;\frac{\sqrt{{\left(2NIR+1\right)}^{2}-8\left(NIR+Red\right)}}{2}$$

Where:

NIR is the reflectance in the near-infrared band
Red is the reflectance in the red band

This formulation of MSAVI automatically adjusts the soil brightness correction factor based on the actual vegetation cover in the scene, thereby improving the accuracy of vegetation monitoring in environments where the soil background can significantly affect the reflectance measurements. Unlike SAVI, which uses a fixed correction factor, MSAVI's dynamic adjustment enables it to provide a more precise vegetation index, especially in heterogeneous landscapes where vegetation cover varies widely. MSAVI is particularly valuable in remote sensing applications for monitoring vegetation health, estimating biomass, and assessing crop productivity, especially in semi-arid and arid regions where the soil exposure is high. By reducing the soil's impact on the vegetation index, MSAVI enhances the sensitivity to vegetation changes, making it a preferred choice for studies that require detailed analysis of vegetation in challenging environments.

This index has been extensively used in agricultural monitoring, environmental studies, and land cover classification, where accurate vegetation assessment is critical. Its ability to dynamically adjust for soil influences makes MSAVI a robust tool for satellite-based vegetation monitoring, providing more reliable data for decision-making in precision agriculture and ecological management. MSAVI further refines SAVI by making the soil brightness correction factor dependent on vegetation cover itself. This index is highly effective in regions with varying soil and vegetation conditions, providing a more accurate assessment of vegetation health [23].

**Modified Chlorophyll Absorption Ratio Index (MCARI)**: MCARI is designed to detect chlorophyll content, which is directly related to the health and density of vegetation. It is particularly effective in scenarios where chlorophyll content is a critical indicator of crop vitality [24]. The Modified Chlorophyll Absorption Ratio Index (MCARI) is a vegetation index designed to enhance the sensitivity of remote sensing measurements to chlorophyll content in vegetation. Chlorophyll content is a critical indicator of plant health, photosynthetic activity, and overall vigour, making it a key parameter in agricultural monitoring, ecological studies, and vegetation assessment.

MCARI was developed to address the limitations of previous indices by reducing the effects of soil background and non-photosynthetic elements, such as stems and dead leaves, on the measurement of chlorophyll content. This index is particularly useful in environments where the soil background significantly influences the reflectance values, potentially leading to inaccuracies in assessing chlorophyll levels using other vegetation indices. The mathematical formula (Eq. 4) is expressed as:

(4) $$MCARI=\;\frac{R_{700}-\;R_{670}}{R_{700}-\;R_{500}}\times \frac{R_{700}}{R_{670}}$$

Where:

R_700_ is the reflectance at 700 nm (a wavelength where chlorophyll absorption is minimal)

R_670_ is the reflectance at 670 nm (a wavelength where chlorophyll absorption is strong)

R_550_ is the reflectance at 550 nm (a wavelength associated with the green reflectance peak)

MCARI is specifically designed to minimize the impact of soil background by using reflectance at 550 nm as a reference for the green peak, which is less influenced by chlorophyll absorption and more affected by non-photosynthetic elements. By focusing on the ratio of reflectance differences between 700 nm and 670 nm, MCARI effectively isolates the chlorophyll absorption feature, providing a more accurate assessment of chlorophyll content.

MCARI has been widely used in precision agriculture, forestry, and environmental monitoring to assess crop health, estimate biomass, and monitor stress levels in plants. Its ability to accurately reflect chlorophyll content, even in the presence of varying soil backgrounds and mixed vegetation types, makes it a valuable tool for remote sensing applications that require precise vegetation monitoring.

#### Modified possibilistic C-means (MPCM) clustering

After calculating the vegetation indices, the Modified Possibilistic C-Means (MPCM) clustering algorithm is employed to classify the vegetation indices into distinct classes. The MPCM algorithm is selected for its ability to handle uncertainty and mixed boundaries by assigning varying degrees of membership to each pixel rather than enforcing a hard classification into a single class [25]. Modified Possibilistic C-Means (MPCM) clustering is an advanced clustering algorithm that builds upon the traditional Possibilistic C-Means (PCM) algorithm, which itself is an extension of the Fuzzy C-Means (FCM) algorithm. The MPCM algorithm is designed to address some of the limitations inherent in PCM, particularly those related to noise sensitivity and the handling of overlapping clusters.

The traditional PCM algorithm, introduced by Krishnapuram and Keller [25], allows each data point to belong to multiple clusters with varying degrees of membership, similar to FCM. However, unlike FCM, PCM does not require the sum of memberships for each data point to be equal to one. Instead, PCM introduces a possibilistic approach where membership values represent the degree of typicality or compatibility of each data point with the cluster rather than its probability. This flexibility makes PCM particularly useful for dealing with data where clusters are not well separated.

Despite its advantages, PCM has some drawbacks, such as sensitivity to initialization and noise, which can lead to coincident clusters where multiple clusters overlap significantly, reducing the algorithm's effectiveness. The Modified Possibilistic C-Means (MPCM) algorithm was developed to mitigate these issues. MPCM incorporates additional constraints and adjustments, such as the integration of spatial information or the inclusion of regularization terms, to enhance the robustness of the clustering process. These modifications help to better manage noise and prevent the formation of coincident clusters, thereby improving the accuracy and reliability of the clustering results in complex datasets.

MPCM is particularly useful in applications where the data contains significant overlap between clusters or where the presence of noise complicates the clustering process. This makes it a powerful tool in fields such as remote sensing, medical imaging, and agricultural data analysis, where accurate classification and segmentation are critical. By incorporating possibilistic principles and modifying the traditional PCM algorithm, MPCM provides a more flexible and robust approach to clustering, allowing for more precise identification of patterns within noisy and overlapping data.

#### Soft classification of vegetation indices

The bands calculated using the vegetation indices (SAVI, MSAVI, MCARI) are then soft classified, meaning that each pixel can belong to multiple classes with varying degrees of membership. This soft classification approach is particularly beneficial in agricultural contexts where boundaries between different vegetation types (e.g., crops, weeds, soil) are not always clear-cut. Traditional hard classification methods may fail to capture the full complexity of these transitions, leading to misclassification [26].

Here, the MPCM clustering algorithm is employed, with an incremental signature addition process. This approach allows for the classification of vegetation indices into distinct classes, where “incremental signature addition” suggests iteratively refining the classification by adding new classes or signatures. The indices are then soft classified, meaning that each pixel can belong to multiple classes with varying degrees of membership, which is useful when class boundaries are not clear-cut. This approach enhances the precision of classification, making it more adaptable to the complex and variable nature of agricultural environments [27].

Finally, the soft-classified bands resulting from SAVI, MSAVI, and MCARI undergo a quantitative assessment. This step likely involves comparing the classification results to ground truth data or other reference datasets to evaluate the accuracy and effectiveness of the classification and the indices used. Overall, this methodology integrates image enhancement, vegetation index calculation, and advanced classification techniques to effectively analyse vegetation from remote sensing data.

#### Quantitative assessment

Finally, a quantitative assessment is performed to evaluate the accuracy and effectiveness of the methodology. This assessment involves comparing the classification output with ground truth data or other reference datasets to determine the performance of the classification algorithm. Metrics such as the Fuzzy Error Matrix (FERM) are used to assess classification accuracy for each class (e.g., soil, weeds, crops), providing a detailed evaluation of the method's effectiveness across different scenarios [28]. The Fuzzy Error Matrix (FERM) is an advanced tool used in the evaluation of classification accuracy, particularly in contexts where fuzzy or soft classification methods are applied. Traditional error matrices, often used in remote sensing and image classification, operate on a crisp classification basis, meaning each pixel is assigned to a single class. In contrast, FERM is designed to handle the complexities of fuzzy classification, where a pixel may belong to multiple classes with varying degrees of membership [29].

FERM extends the traditional confusion matrix by incorporating the membership values of pixels across different classes, rather than relying on a binary (correct/incorrect) assessment. This allows for a more nuanced evaluation of classification performance, particularly in situations where class boundaries are not well defined, or where there is significant overlap between classes, such as in the classification of soil, weeds, and crops in agricultural fields. The construction of FERM involves comparing the fuzzy classification results to reference data, which may also be represented in fuzzy terms. For each class, the matrix records the degree of membership that the classified pixels have in the reference classes. This approach allows for a comprehensive evaluation of how well the classification process reflects the true distribution of classes in the study area.

In the context of mapping the classification accuracy of soil, weeds, and crops, FERM is particularly useful. For instance, in an agricultural landscape where there may be a gradual transition between soil, weed-infested areas, and healthy crops, FERM can capture the partial memberships of pixels in these overlapping regions. This is critical in precision agriculture, where management decisions often depend on accurate and detailed maps that reflect the complexity of the field conditions. By using FERM, researchers and practitioners can obtain a more accurate assessment of classification performance. This includes identifying areas where the classification is uncertain or where the model might be confusing different classes. For example, if a particular area of a field is misclassified due to the spectral similarity between weeds and crops, FERM can help quantify the degree of this misclassification, providing insights into how the classification model might be improved. FERM is, therefore, an essential tool in any application where fuzzy classification is employed, enabling a deeper understanding of classification accuracy and supporting the development of more reliable classification models.

##### Soft-classified CARI bands

**Fig. 2:**
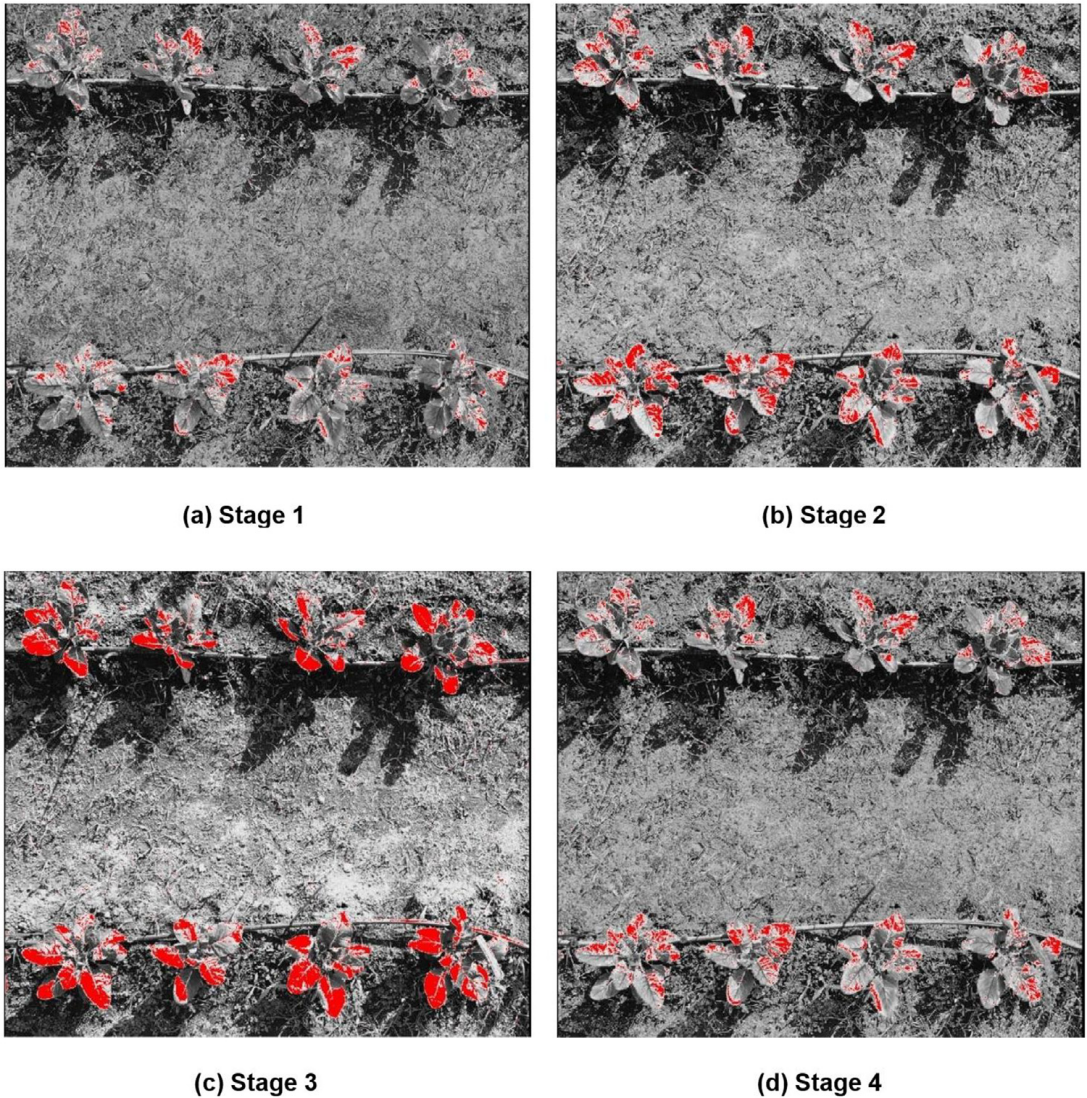
Chlorophyll absorption ratio index + MPCM soft classified applied on crop images with varyinginputs of manual signatures **(a)** 24 manual signatures **(b)** 36 manual signatures **(c)** 48 manual signatures **(d)** 60 manual signatures.

The Fuzzy Error Matrix for soft classified MCARI bands representing soil, weeds and crops classes are described in Table 1. The best classification was observed in stage 3 (Fig. 2(c)) where 48 manual signatures were taken over crop leaves. It was also observed that on soft classification performed with 60 manual signature points led to misclassification. This is particularly due to spectral overlap among weed and crop classes. The Fuzzy Error Matrix (FERM) for crop classification using MCARI and MPCM reveals varied performance across different classes. The classification accuracy for crops is the highest, with 73% of crop instances correctly identified, indicating effective feature separation for crops. However, soil classification shows poor accuracy, with only 32% correctly classified, and significant misclassification as weeds (34%) and crops (34%). Weeds are moderately classified with 57% accuracy but still exhibit notable confusion with soil (21%) and crops (22%). These results suggest that while MCARI and MPCM effectively distinguish crops, they struggle with soil and weeds, highlighting the need for additional spectral indices or features and potential algorithm tuning to enhance overall classification accuracy and reduce misclassification.

**Table 1:** Fuzzy error matrix for best stage Fig 2(c) MPCM soft classified CARI.

FERM	Predicted Soil	Predicted Weeds	Predicted Crops
True Soil	0.32	0.34	0.34
True Weeds	0.21	0.57	0.22
True Crop	0.13	0.14	0.73

#### Soft-classified MSAVI bands

**Fig. 3:**
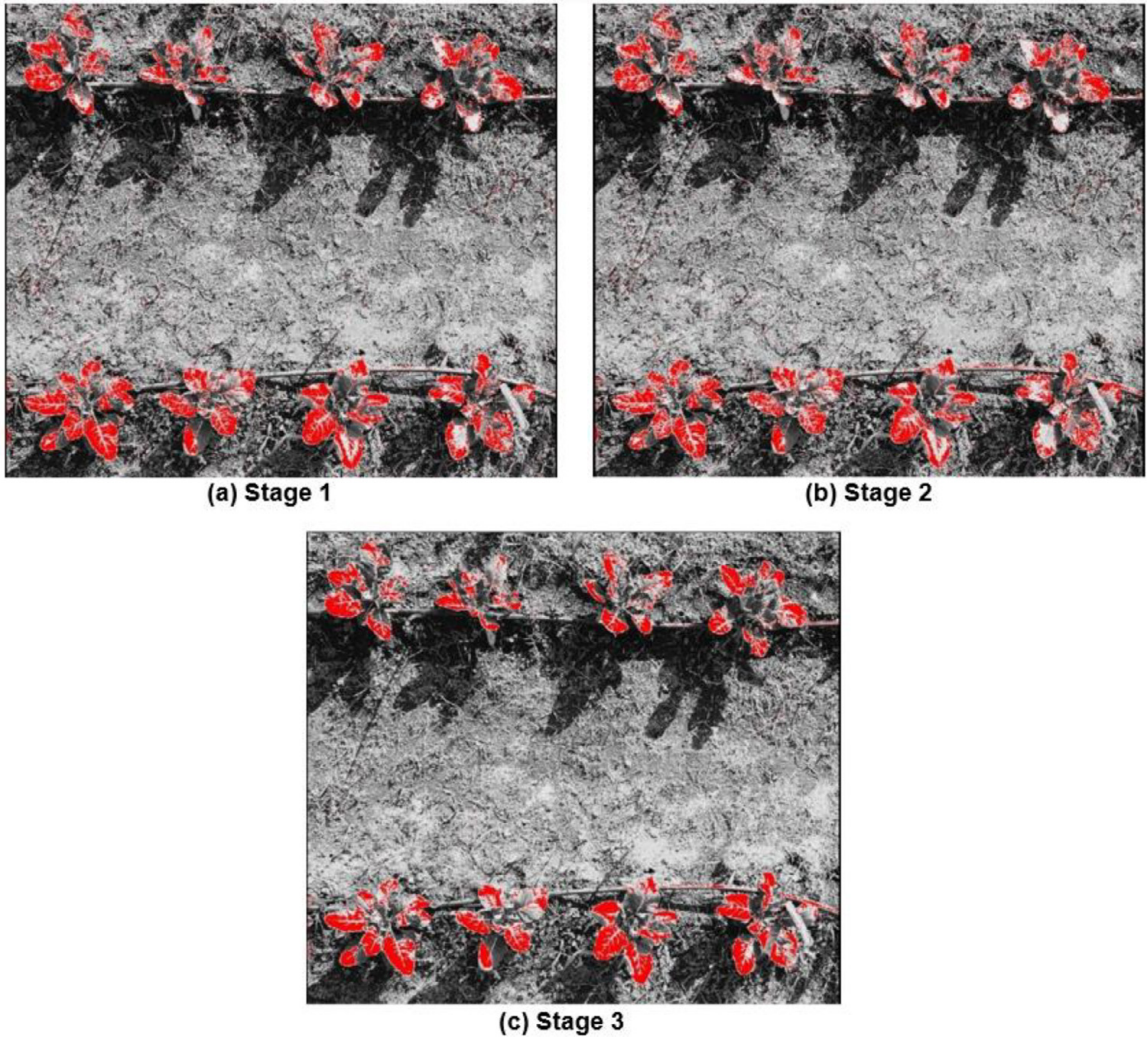
Modified soil adjusted vegetation index + MPCM soft classified applied on crop images with varying inputs of manual signatures **(a)** 24 manual signatures **(b)** 36 manual signatures, **(c)** 48 manual signatures.

The Fuzzy Error Matrix for soft classified MSAVI bands representing soil, weeds and crops classes are described in Table 2. The best classification was observed in stage 3 (Fig. 3(c)) where 48 manual signatures were taken over crop leaves. It was observed to have an incremental classification accuracy linked with addition of more manual signatures for the crop classification. This is particularly due to spectral overlap among weed and crop classes. The Fuzzy Error Matrix (FERM) for crop classification using MSAVI and MPCM reveals incremental performance across different classes. The Fuzzy Error Matrix (FERM) for crop classification using MSAVI and MPCM reveals that the classification system is most effective for crops, with an impressive 85% of true crop instances correctly identified as predicted crops. In comparison, the classification accuracy for weeds is moderate, with 48% of true weed instances accurately classified, although there is still notable misclassification with 12% being identified as soil and 40% as crops. Soil classification exhibits the lowest accuracy, with only 18% of true soil instances correctly identified, while a significant 27% are misclassified as weeds and 55% as crops. These results indicate that while the system excels at identifying crops, there is considerable room for improvement in accurately distinguishing soil and weeds, highlighting the need for additional features or algorithm tuning to enhance overall classification performance.

**Table 2:** Fuzzy error matrix for best stage Fig 3(c) MPCM soft classified MSAVI.

FERM	Predicted Soil	Predicted Weeds	Predicted Crops
True Soil	0.18	0.27	0.55
True Weeds	0.12	0.48	0.40
True Crop	0.04	0.11	0.85

##### Soft-classified SAVI bands

**Fig. 4:**
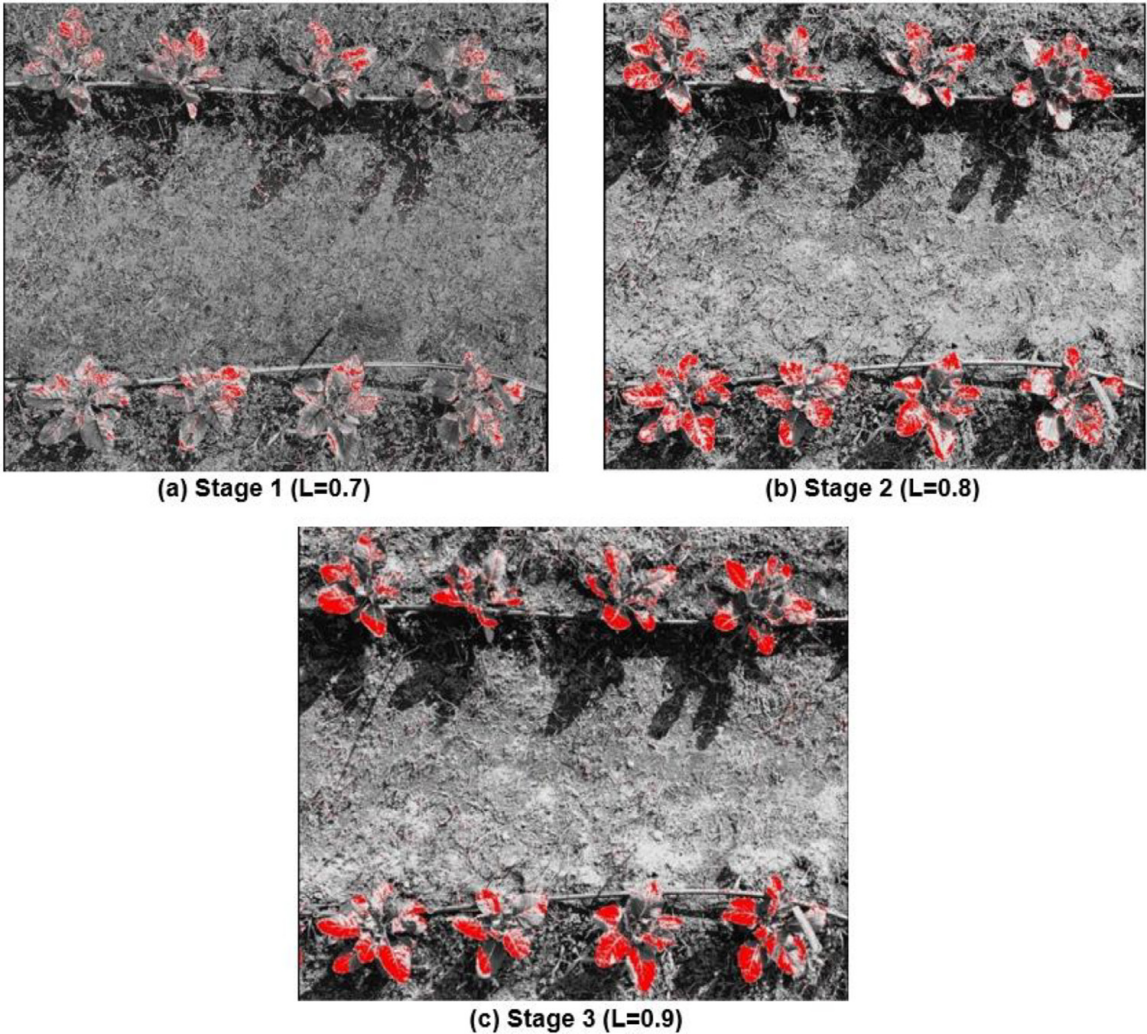
Soil adjusted vegetation index + MPCM soft classified applied on crop images with varying inputs of soil brightness reflectance factor (L): **(a)** L=0.7 **(b)** L=0.8 **(c)** L=0.9.

The best results for MPCM soft classified SAVI were achieved with a soil brightness correction value, L of 0.9, providing a balance between accuracy and minimal misclassification among soil, weeds, and crops (Table 3; Fig. 4(c)). The Root Mean Square Error (RMSE) for SAVI was moderately low, indicating reasonable precision, though not as precise as the results obtained using MSAVI. The Fuzzy Error Matrix showed that 18% of true soil instances were correctly classified, 50% of true weeds were accurately identified, and 86% of true crops were correctly classified, making SAVI's performance superior to that of MCARI, which struggled with higher misclassification rates and a lower accuracy of 75% for true crops.

**Table 3:** Fuzzy error matrix for best stage Fig 4(c) MPCM soft classified SAVI.

FERM	Predicted Soil	Predicted Weeds	Predicted Crops
True Soil	0.18	0.22	0.60
True Weeds	0.12	0.50	0.38
True Crop	0.04	0.10	0.86

### Method validation

**Fig. 5:**
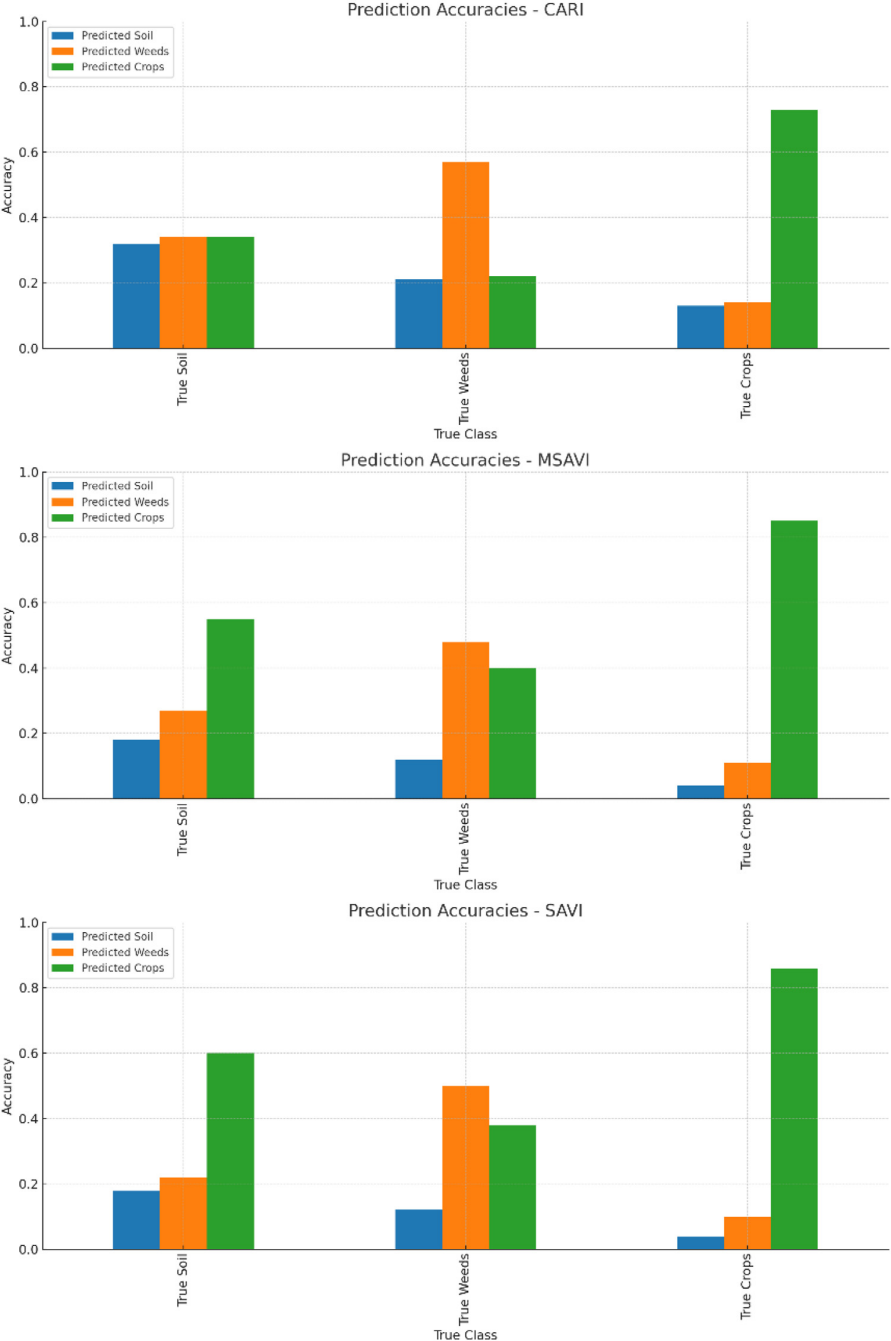
Prediction accuracies of soft classified CARI, MSAVI and SAVI.

The CARI index demonstrates a strong performance in detecting crops, achieving an accuracy of 73% (Fig. 5). On the other hand, the MSAVI index excels in crop detection, boasting the highest crop prediction accuracy of 85%. Similarly, the SAVI index also shows excellent performance in crop prediction, with an accuracy of 86%, making it the most accurate among the three indices. Both MSAVI and SAVI share the strength of high crop detection accuracy, with SAVI having a slight edge over MSAVI.

### Comparison of prediction accuracies

The set of bar charts compares the prediction accuracies for soil, weeds, and crops across the three indices (Fig. 5). It clearly shows that CARI has a balanced but lower performance in predicting all three classes. MSAVI and SAVI excel in crop detection, with SAVI having a slight edge. CARI performs better in weed detection compared to SAVI and MSAVI.

### Confusion Matrix and combined performance in classification

**Fig. 6:**
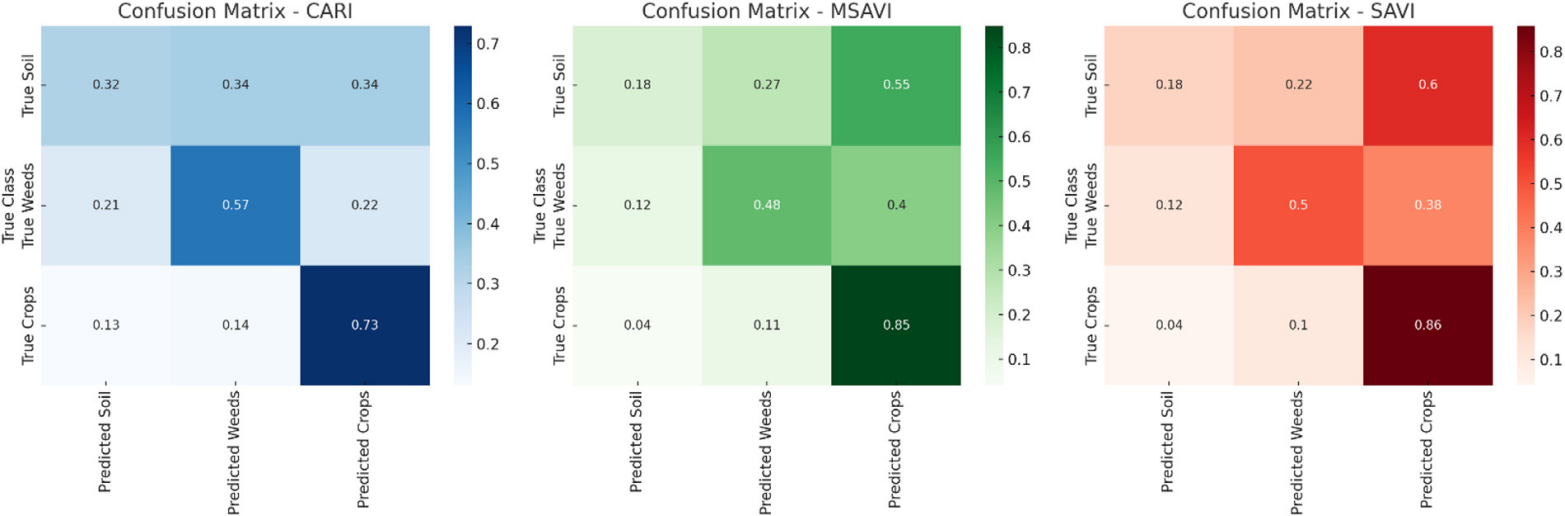
Confusion matrix heatmaps of soft classified CARI, MSAVI and SAVI.

**Fig. 7:**
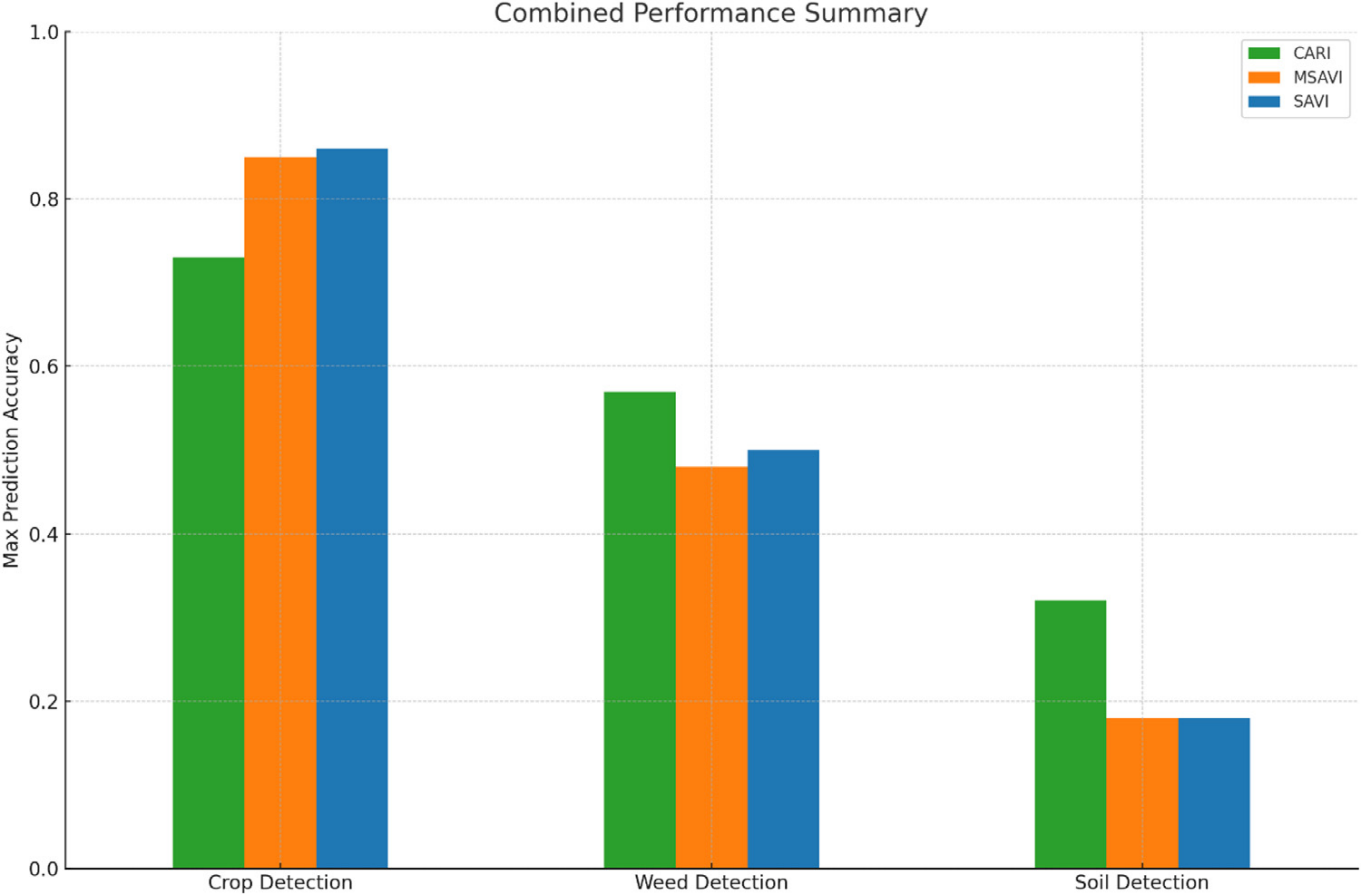
Overall performance of soft-classified MCARI, MSAVI and SAVI.

The confusion matrix heatmaps shows CARI with higher misclassification rates between soil, weeds, and crops (Fig. 6, Fig. 7). MSAVI and SAVI show stronger performance in crop detection, with fewer misclassifications. SAVI emerges as the best overall performer, especially for crop detection, followed closely by MSAVI. CARI remains competitive in weed detection, outperforming the others in this specific area.

### Limitations

Combining vegetation-based spectral indices with fuzzy-based algorithms presents additional challenges in classifying and segmenting crops and weeds, particularly in heterogeneous agricultural environments. One significant issue is the spectral similarity between crops and weeds, which often leads to classification inaccuracies. Mixed pixels containing both crop and weed information exacerbate this problem, reducing the effectiveness of fuzzy-based algorithms in clearly distinguishing between the two. Additionally, varying environmental conditions such as different lighting, soil backgrounds, and growth stages introduce variability that affects the accuracy of spectral indices. This variability challenges fuzzy algorithms to maintain consistent performance across different scenarios [[1], [2], [3], [4]].

Occlusion and overlapping of plant leaves, common in dense vegetation, lead to significant difficulties in distinguishing individual plants. This complicates the segmentation process as fuzzy algorithms may misclassify occluded or overlapping areas as a single entity [[5], [6], [7], [8]]. Furthermore, the high dimensionality of hyperspectral data, used alongside fuzzy algorithms, increases computational complexity, making real-time applications challenging due to the significant computational resources required [9,10].

Selecting the most relevant spectral indices and tuning the parameters of fuzzy algorithms to achieve optimal performance is another complex task. Different indices and parameters yield varying results depending on the specific agricultural context, necessitating extensive experimentation and calibration [[11], [12], [13]]. Additionally, efficiently combining spatial and spectral information to enhance classification accuracy presents a significant challenge. While spatial information helps distinguish different vegetation structures, integrating it with spectral data in a fuzzy framework requires sophisticated algorithms and precise calibration [14,15].

In conclusion, while the combination of vegetation-based spectral indices with fuzzy-based algorithms holds promise for crop and weed classification and segmentation, several challenges remain. Addressing issues of spectral similarity, environmental variability, occlusion, high dimensionality, optimal index selection, and spatial-spectral integration requires further research and development of more robust, adaptive algorithms tailored to specific agricultural applications [[16], [17], [18], [19]].

## Data availability

https://data.mendeley.com/datasets/dcjjcwc5dh/3

## Ethics Statements

This research did not involve human subjects, animal experiments, or data collected from social media platforms. Therefore, no specific ethical approvals or informed consent were required for this study.

## CRediT authorship contribution statement

**Shubham Rana**: Conceptualization, Methodology, Validation, Data curation, Writing-Original draft preparation,Editing; **Salvatore Gerbino**: Supervision, Reviewing; **Petronia Carillo**: Supervision, Reviewing.

## Declaration of competing interest

The authors declare that they have no known competing financial interests or personal relationships that could have appeared to influence the work reported in this paper.
